# Reference range of complete blood count, Ret-He, immature reticulocyte fraction, reticulocyte production index in healthy babies aged 1–4 months

**DOI:** 10.1038/s41598-023-27579-3

**Published:** 2023-01-09

**Authors:** Harapan Parlindungan Ringoringo, Lina Purnamasari, Ari Yunanto, Meitria Syahadatina, Nurul Hidayah

**Affiliations:** 1 Division of Hematology-Oncology, Department of Child Health, Faculty of Medicine, Lambung Mangkurat University – RSD Idaman Banjarbaru, Banjarbaru, South Kalimantan Indonesia; 2 Pediatric Resident, Department of Child Health, Faculty of Medicine, Lambung Mangkurat University- Ulin General Hospital, Banjarmasin, South Kalimantan Indonesia; 3 Division of Nenonatology, Department of Child Health, Faculty of Medicine, Lambung Mangkurat University- Ulin General Hospital, Banjarmasin, South Kalimantan Indonesia; 4 Faculty of Medicine, Reproductive Health-Maternal and Child Health-Family Planning, Lambung Mangkurat University, Banjarmasin, South Kalimantan Indonesia; 5 Division of Neurology, Department of Child Health, Faculty of Medicine, Lambung Mangkurat University- Ulin General Hospital, Banjarmasin, South Kalimantan Indonesia

**Keywords:** Biomarkers, Diseases, Medical research, Neurology

## Abstract

Establishing reference ranges of the complete blood count (CBC), reticulocyte hemoglobin content (Ret-He), immature reticulocyte fraction (IRF), and reticulocyte production index (RPI) helps diagnose a disease related to the changes in erythrocyte indices, white blood count, platelets, and reticulocytes, especially in babies. Therefore, the study aims to establish a reference range for CBC and reticulocyte parameters in healthy babies aged 1–4 months. The study design was a cross-sectional study with descriptive analysis of CBC and reticulocyte in babies aged 1–4 months. Three hundred forty-eight babies met the inclusion criteria. This study recruited 89 babies aged 1 month, 87 babies aged 2 months, 86 babies aged 3 months, and 86 babies aged 4 months. The P5-P95 reference range of healthy babies for hemoglobin (Hb) aged 1 month, 2 months, 3 months, and 4 months was 9.95 to 15.45 g/dL, 9.74 to 13.42 g/dL, 9.51 to 12.40 g/dL, and 10.04 to 13.10 g/dL respectively. The P3-P97 reference range of healthy babies for Hb aged 1 month, 2 months, 3 months, and 4 months was 9.60 to 15.90 g/dL, 9.46 to 13.97 g/dL, 9.26 to 12.82 g/dL, and 10.00 to 13.33 g/dL respectively. This study also defined reference ranges for CBC, Ret-He, IRF, and RPI. The reference range of CBC, Ret-He, IRF, and RPI for healthy babies aged 1–4 months in this study can be used as a benchmark.

## Introduction

All ages anemia prevalence was 22.8% globally in 2019. The prevalence was highest among children under five years, 39.7%, with the most contributing cause being dietary iron deficiency (ID)^1^. Anemia is still a major global health problem. Thereby, the early detection of anemia is crucial considering its long-term effects that can interfere with a baby’s growth and development. Several studies have shown the impact of iron deficiency anemia (IDA) on cognitive and behavioral deteriorations^2–4^. Then, early diagnosis and treatment of impending brain dysfunction in the pre-anemic stage are necessary to prevent neurological deficits. Iron deficiency and IDA are caused by not giving oral iron supplementation and low iron stores at birth; the baby’s growth rate is swift, and inadequate iron intake.

Reference range data for complete blood count (CBC), reticulocyte hemoglobin content (Ret-He), immature reticulocyte fraction (IRF), and reticulocyte production index (RPI) for babies 1–4 months old are limited. Therefore, determination of the reference range of CBC, Ret He, IRF, and RPI in babies 1–4 months is essential to determine whether a baby is anemic or not. Combining several parameters, Hb (hemoglobin), MCV (mean corpuscular volume), MCH (mean corpuscular hemoglobin), RDW (red cell distribution with), reticulocyte count, Ret He, IRF, and RPI will undoubtedly make it easier to determine if a baby has ID before IDA occurs. In addition, to monitor oral iron therapy, these combining parameters can be used too. For example, a lower value than the lower limit of the Hb reference range according to age indicates a baby is anemic.

On the other hand, other erythrocyte indices reference ranges are needed to diagnose anemia, especially suspected IDA. The main problem is determining if a baby has IDA with a simple, cheap, and fast laboratory test; a small amount of blood is drawn and can be done in almost any health facility. It is even better if the ID can be detected before IDA occurs.

Conventionally, ID diagnosis is based on determining biochemical parameters, such as ferritin, transferrin saturation, and soluble transferrin receptor. Nevertheless, these biomarkers are strongly influenced by the diurnal phase, inflammation, infection, and malignancy, and of course, they are expensive, and not every health facility can check them. In addition, many studies have shown that Ret-He can show iron deficiency without anemia^5–7^. Therefore, a lower value than the lower limit of the Ret-He reference range according to age indicates that an infant is iron deficient.

The clinical utility of IRF applied in various circumstances, such as monitoring anemia treatment and neonatal transfusion needs, can be a prognostic factor in prematurity anemia and diagnosing and monitoring aplastic anemias. Furthermore, RPI monitors bone marrow response to iron-treated anemia^8^. In addition, RPI can be used as a diagnostic guide to the etiology of anemia^9^. Uniquely, all erythrocyte indices and reticulocyte parameters can be seen automatically on the Sysmex XN-450 Hematology Analyzer (Sysmex Corporation, Japan).

The purpose of this study was to obtain the lower limit of the baby's hemoglobin value related to the age so that it can be used to declare an anemic baby or not. Moreover, establishing reference ranges of the CBC, Ret-He, IRF, and RPI helps diagnose a disease related to the changes in erythrocyte indices, white blood count, platelets, and reticulocytes, especially in healthy babies aged 1–4 months.

## Results

Three hundred forty-eight healthy babies (boys 53.4%, girls 46.6%) met the inclusion criteria. Therefore, this study recruited 89 babies aged 1 month (boys 59.6%, girls 40.4%), 87 babies aged 2 months (boys 49.4%, girls 50.6%), 86 babies aged 3 months (boys 52.3%, girls 47.7%), and 86 babies aged 4 months (52.3% boys, 47.7% girls). P5-P95 and P3-P97 of the complete blood count, reticulocyte count, Ret-He, immature reticulocyte fraction, and reticulocyte production index for healthy babies aged 1–4 months can be seen in Tables 1 and 2.

**Table 1:** P5-P95 of the white blood count, red blood cell count, hemoglobin, hematocrit, mean corpuscular volume, mean corpuscular hemoglobin, mean corpuscular hemoglobin concentration, platelet, red cell distribution with standard deviation, red cell distribution with coefficient of variation, platelet distribution with, mean platelets volume, plateletcrit, neutrophils, lymphocyte, monocyte, eosinophils, eosinophils absolute, basophils, basophils absolute, reticulocyte count, Ret-He, immature reticulocyte fraction, reticulocyte production index, immature platelet fraction, and immature granulocyte for healthy babies aged 1–4 months.

CBC	1 Month (N = 89)	2 Months (N = 87)	3 Months (N = 86)	4 Months (N = 86)
WBC (10ˆ3/uL)	6.24–12.76	5.73–12.84	6.88–13.66	6.06–14.32
RBC (10ˆ6/uL)	3.03–4.89	3.21–4.55	3.38–4.70	3.71–5.09
HGB (g/dL)	9.95 – 15.45	9.74–13.42	9.51–12.40	10.04–13.10
HCT (%)	28.20–43.95	28.00–38.14	28.14–35.80	29.14–38.36
MCV (fL)	86.35–101.85	78.54–95.42	71.91–88.33	70.25–84.85
MCH (pg)	29.75–35.20	27.48–32.72	24.70–30.27	23.64–28.97
MCHC (g/dL)	32.25–36.90	32.78–37.00	32.84–36.27	32.07–35.50
PLT (10ˆ3/ul)	0.13–0.57	0.07–0.63	0.10 – 0.62	0.16–0.59
RDW SD (fL)	43.25–64.50	35.54–52.36	33.00–44.94	30.90–39.69
RDW CV (%)	13.05–18.30	11.94–15.54	11.54–14.93	11.50–13.96
PDW(fL)	9.50–16.97	8.43–19.12	8.62–15.94	9.23–16.42
MPV(fL)	9.30–12.47	8.90–12.67	8.80–12.06	9.09–12.31
PCT (%)	0.19–0.60	0.16–0.63	0.29–0.62	0.19–0.60
Neutrophils (Gpt/L)	0.81–3.55	0.77–4.62	1.07–3.95	0.85–4.54
Lymphocyte (Gpt/L)	3.80–7.68	3.88–8.48	4.33–9.50	4.13–10.16
Monocyte Gpt/L)	0.61–1.86	0.41–1.37	0.42–1.42	0.33–1.19
Eosinophils (Gpt/L)	0.22–1.31	0.14–0.79	0.14–1.11	0.16–1.26
Eosinophils absolut (Gpt/L)	1.45–13.64	0.93–8.96	1.13–13.05	1.12–17.47
Basophils (Gpt/L)	0.02–0.13	0.01–0.11	0.02–0.16	0.01–0.15
Basophils absolut (Gpt/L)	0.15–1.34	0.10–1.05	0.14–1.73	0.09–1.82
Reticulocyte (Tpt/L)	5.95–22.35	4.38–21.64	3.00–16.91	2.94–12.87
Reticulocyte (%)	0.47–2.06	0.92–3.11	0.63–2.90	0.56–1.78
Ret_He (pg)	21.95–28.30	20.74–26.60	17.96–29.70	19.22–29.70
IRF(%)	5.95–22.35	4.38–21.64	3.00–16.91	2.94–12.87
RPI	0.30–1.10	0.34–1.42	0.24–1.30	0.20–0.90
IPF (%)	0.60–15.80	0.54–16.58	0.60–12.56	0.74–13.20
IG (Gpt/L)	0.01–0.12	0.00–0.09	0.00–0.04	0.00–0.03

**Table 2:** P3-P97 of the white blood count, red blood cell count, hemoglobin, hematocrit, mean corpuscular volume, mean corpuscular hemoglobin, mean corpuscular hemoglobin concentration, platelet, red cell distribution with standard deviation, red cell distribution with coefficient of variation, platelet distribution with, mean platelets volume, plateletcrit, neutrophils, lymphocyte, monocyte, eosinophils, eosinophils absolute, basophils, basophils absolute, reticulocyte count, Ret-He, immature reticulocyte fraction, reticulocyte production index, immature platelet fraction, and immature granulocyte for healthy babies aged 1–4 months.

CBC	1 Month (N = 89)	2 Months (N = 87)	3 Months (N = 86)	4 Months (N = 86)
WBC (10ˆ3/uL)	5.88–13.43	5.46–13.85	6.55–16.14	5.86–15.16
RBC (10ˆ6/uL)	2.92–5.09	3.08–4.62	3.08–4.78	3.54–5.15
HGB (g/dL)	9.60–15.90	9.46–13.97	9.26–12.82	10.00–13.33
HCT (%)	27.13–48.32	26.95–39.71	27.57–36.69	28.62–39.04
MCV (fL)	84.28–103.63	77.73–96.82	70.35–90.94	65.25–86.62
MCH (pg)	29.17–35.65	26.86–33.02	24.03–30.99	22.48–29.10
MCHC (g/dL)	31.67–37.29	32.49–37.50	32.02–36.38	31.92–36.04
PLT (10ˆ3/ul)	0.09–0.64	0.05–0.65	0.10–0.66	0.15–0.61
RDW SD (fL)	42.44–67.15	35.12–55.56	32.56–48.59	30.70–40.60
RDW CV (%)	12.97–18.96	11.60–16.10	11.20–17.35	11.42–14.41
PDW(fL)	8.82–17.84	8.20–22.81	8.45–16.10	8.93–17.50
MPV(fL)	8.90–12.58	8.70–13.20	8.70–12.50	8.77–12.64
PCT (%)	0.16–0.67	0.15–0.66	0.12–0.65	0.18–0.65
Neutrophils (Gpt/L)	0.74–4.08	0.66–5.34	1.00–4.97	0.77–5.08
Lymphocyte (Gpt/L)	3.30–8.12	3.46–8.95	4.11–10.42	3.48–10.73
Monocyte Gpt/L)	0.60–2.06	0.35–1.43	0.41–1.50	0.31–1.41
Eosinophils (Gpt/L)	0.19–1.50	0.12–0.84	0.11–1.17	0.15–2.17
Eosinophils absolut (Gpt/L)	1.00–14.61	0.85–9.54	1.03–15.00	0.78–27.34
Basophils (Gpt/L)	0.02–0.13	0.01–0.14	0.02–0.23	0.01–0.17
Basophils absolut (Gpt/L)	0.14–1.48	0.05–1.16	0.12–3.02	0.07–2.35
Reticulocyte (Tpt/L)	5.61–25.58	3.70–23.12	2.67–25.66	2.33–13.38
Reticulocyte (%)	0.38–2.53	0.58–3.49	0.57–3.32	0.52–1.98
Ret_He (pg)	21.74–28.56	19.85–27.24	16.78–30.24	17.40–30.22
IRF(%)	5.61–25.58	3.70–23.12	2.67–25.66	2.33–13.38
RPI	0.20–1.48	0.26–1.60	0.20–1.40	0.20–1.00
IPF (%)	0.57–18.48	0.50–20.79	0.50–17.04	0.70–14.38
IG ( Gpt/L )	0.01–0.14	0.00–0.14	0.00–0.04	0.00–0.03

The P5-P95 reference range of healthy babies for hemoglobin (Hb) aged 1 month, 2 months, 3 months, and 4 months was 9.95 to 15.45 g/dL, 9.74 to 13.42 g/dL, 9.51 to 12.40 g/dL, and 10.04 to 13.10 g/dL respectively. The P3-P97 reference range of healthy babies for Hb aged 1 month, 2 months, 3 months, and 4 months was 9.60 to 15.90 g/dL, 9.46 to 13.97 g/dL, 9.26 to 12.82 g/dL, and 10.00 to 13.33 g/dL respectively.

## Discussion

Establishing the lower limit of P3-P97 and P5-P95 on examining specific parameters is commonly used statistically. For example, the WHO (World Health Organization) and CDC (Centers Of Disease Control and Prevention) growth charts set the lower limit of Weight-for-age, Length/height-for-age, Head circumference-for-age, and Arm circumference-for-age at P3^10^. Determination of the lower limit of Hb with P3-P97 and P5-P95, of course, by considering race, ethnicity, age, gender, and altitude above sea level^11^. This study accommodates all reference range limits to be used flexibly.

The results of this study showed the reference range of CBC, reticulocyte count, Ret-He, IRF, and RPI at P3-P97 and P5-P95. Values below the lower limit of the reference range of Hb according to age can be used as a limit to determine if a baby has anemia. In addition, the value below the lower limit of the reference range of Ret-He according to age can be used as a limit to determine whether a baby has ID. The baby is suffered from IDA; if the value of Hb and Ret-He is below the lower limit of the reference range. Thus, for example, this study indicates that the lower limit of Hb for healthy babies aged 4 months (see Tables 1 and 2) can be taken at P3 and P5, i.e., 10.00 g/dL and 10.04 g/dL, respectively. In addition, this study indicates that the lower limit of Ret-He for healthy babies aged 4 months (see Tables 1 and 2) can be taken at P3, and P5, i.e., 17.40 pg and 19.22 pg, respectively.

Therefore, to determine whether a baby aged 4 months is suffering from IDA if the value of Hb and Ret-He is below the lower limit of the reference range. Combining several parameters, Hb, MCV, MCH, RDW, reticulocyte count, and Ret He, in conjunction with the peripheral blood smear examination, will make an IDA diagnosis undoubtedly. The examples above can also be used for babies aged 1 month, 2 months, and 3 months (see Tables 1 and 2).

One study showed that IDA, iron depletion and iron deficiency incidence in babies aged 0–6 months were 40.8, 28.0 and 27.0%, respectively. The incidence of IDA in babies aged 0 months, 1 month, and 2 months were 11.8, 10.9 and 11.3%, respectively. This means that the age of 0–6 months, especially 0–2 months, is critical for a baby to suffer from an iron deficiency with or without anemia. Nevertheless, the impact will also affect the incidence of iron deficiency at a later age. Li et al. showed that of 1,127 6-months-old infants in Beijing, the prevalence of anemia was ~ 11.8%^12^. Chen et al. showed that from 509 infants aged 1–12 months, The prevalence of ID and IDA were 3.7 and 2.7%, respectively, in babies under 6 months of age, but increased to 20.4 and 6.6%, respectively, in babies above 6 months of age^13^. Salah et al. show that from 654 infants aged 9–12 months, the prevalence of anemia and IDA was 34.6 and 32.6%, respectively^14^. Nazari et al. found that the prevalence of ID and IDA in children under six years of age was 27.7 and 18.2%, respectively. Considering the high prevalence of ID and IDA in infancy, especially at 0–6 months, iron supplementation in the form of elemental iron at a dose of 1 mg/kg/day should be given to all babies born at term from birth^15^. Another study showed that Daily iron supplementation from early life 36 h at a dose of 2 mg/kg is efficacious for improving iron status and motor development at 6 months in babies at risk^16^.

The results of this study for reference range of other erythrocyte indices, leukocyte, diff count, and platelets can be considered a guide in diagnosing a disease relating to the changes in erythrocyte indices, white blood count, platelets, especially in babies.

Immature reticulocyte fraction indicates the younger fraction of reticulocytes, reflecting erythropoietic activity. Several studies have demonstrated the clinical utility of IRF. For example, an increase in IRF within a few days indicates recovery of bone marrow following bone marrow transplantation, erythropoiesis-stimulating agent therapy, or chemotherapy^17–19^. Molina et al. consider an IRF value greater than 10% to indicate early marrow recovery^20^. Chang et al. stated that increased IRF (IRF ≥ 0.23) and increased absolute reticulocyte count (ARC) generally indicated an adequate erythroid response to anemia. Meanwhile, an IRF of 0.23 or less in patients with anemia reflects bone marrow that is nonresponsive or under-responsive to the anemia^21^. Immature reticulocyte fraction may be the first sign of hematologic recovery. It is a powerful indicator of post-chemotherapy aplasia in children with cancer, serving as an additional parameter of impaired bone marrow function^22^. A low total count with a relatively high IRF indicates a regenerating marrow, whereas reticulocytopenia with low IRF is typical of severe aplastic anemia or renal failure. A high total count with high IRF occurs in acute hemolysis and blood loss. In contrast, a low to average total count with a high IRF occurs in dyserythropoietic and during the early response to haematinics. It may also help decide whether macrocytic anemia is megaloblastic or nonmegaloblastic^23^. Mullier et al. showed that IRF, combined with reticulocyte count, may be helpful in hereditary spherocytosis (HS) diagnosis as a high reticulocyte count characterizes HS without an equally elevated IRF. Therefore, a reticulocytes/IRF ratio higher than 7.7 is a precondition for screening HS cases^24^. The IRF increases earlier than the reticulocyte number. It helps monitor the efficacy of therapy in nutritional anemias such as megaloblastic or IDA. Therefore, the reference value of the IRF range in this study can be considered to assess the responsiveness of the anemia therapy given.

The reticulocyte count reflects the erythropoietic activity of bone marrow. In addition, it is valuable in diagnosing anemias and monitoring bone marrow response to therapy. RPI is currently used in pediatrics to assess bone marrow responsiveness to anemia therapy given. However, considering that “conventional” RPI was developed from a study conducted in adults, it is necessary to have an RPI based on standard values adjusted for different pediatric ages, especially in babies. Therefore, the reference value of the RPI range in this study can be considered to assess the responsiveness of the anemia therapy given. However, according to Bracho et al., the RPI is an inadequate tool for evaluating the bone marrow response in the presence of anemia due to differences in hematologic values between children and adults. In addition, the absence of information on the maturation time of reticulocytes in children^25^.

The power of this study is that it parades all reference ranges of CBC, Ret He, IRF, and RPI at P3-P97 and P5-P95 for healthy babies aged 1–4 months. The weakness of this study is that the altitude above sea level, race, genetics, ethnicity, and population of this study is different from other areas. Hence, it certainly affects the reference range of CBC and reticulocyte parameters. In this study, 96.5% reside at altitudes less than 100 m. Again, however, race, genetics, and ethnicity differed from other countries. Therefore, this reference range for CBC and reticulocyte parameters in healthy babies aged 9–11 months can be used in countries with demographic, socioeconomic, and population structures, such as Southeast Asia, especially Indonesia.

## Material and methods

### Study population

The study design was a cross-sectional study with descriptive analysis of CBC and reticulocyte in babies aged 1–4 months. The study was conducted at 10 Community Health Centers in Banjarbaru, South Kalimantan, from August 2020 to August 2021. Inclusion criteria are babies born at term (gestational age 37–42 weeks) with birth weight ≥ 2500 g, babies are not twins, not taking a hematinic drug, and normal nutritional status. Exclusion criteria were hematological diseases and congenital anomalies. In addition, gestational age and birth weight had obtained from the mother’s medical record (Maternal and Child Health Book). The doctor in charge declared the baby healthy. At the time of recruitment, the baby’s weight, length, and head circumference were measured by health personnel. Nutritional status is assessed based on body weight and length, divided into good nutrition and undernutrition. Good nutrition if the z-score is − 2 SD to + 3 SD in this study. In contrast, undernutrition if the z-score is < − 2 SD^26^. All of the baby’s parents had signed the informed consent. This study obtained ethical clearance from the Research Ethics Commission of the Medical Faculty of the University of Lambung Mangkurat No. 272/KEPK-FK ULM/EC/VIII/2020. All methods have been performed under the relevant guidelines and regulations of the Declaration of Helsinki.

### Blood sampling

Every baby who meets the inclusion and exclusion criteria will be taken a blood sample of 1 ml from the median cubital vein. First, the blood sample was put in a tube with EDTA anticoagulant, homogenized by turning it over, and stored in a storage box. Then the blood sample was sent to the Banjarbaru Idaman Hospital Laboratory. The Sysmex XN-450 Hematology Analyzer (Sysmex Corporation, Japan) was doing complete blood count and reticulocyte examinations.

### Statistical analysis

All baby anthropometry measurements were analyzed by SPSS ver.25 for P3-P97 and P5-P95. Moreover, the CBC and reticulocyte laboratory findings were analyzed by SPSS ver.25 for P3-P97 and P5-P95. All data are presented in narrative and table. Reference range P3-P97 means that 94% of normal individuals have normal laboratory results, while the other 6% may not be sick outside the normal limits. Reference range P5-P95 means that 90% of normal individuals have normal laboratory results, while 10% may not be sick outside the normal limits.

## Supplementary Information

Supplementary Table 1.

Supplementary Table 2.

Supplementary Table 3.

Supplementary Table 4.

Supplementary Table 5.

Supplementary Table 6.

Supplementary Table 7.

## Data Availability

Research data can be provided by the Corresponding Author if needed.
