# Supplementary material for: Reference range of complete blood count, Ret-He, immature reticulocyte fraction, reticulocyte production index in healthy babies aged 1–4 months
Source: Sci Rep. 2023 Jan 9;13:423. doi: 10.1038/s41598-023-27579-3 (PMC9829736; doi:10.1038/s41598-023-27579-3)
Supplement: Supplementary file 1 — Supplementary Table 1. [file 41598_2023_27579_MOESM1_ESM.pptx]

## Slide 1
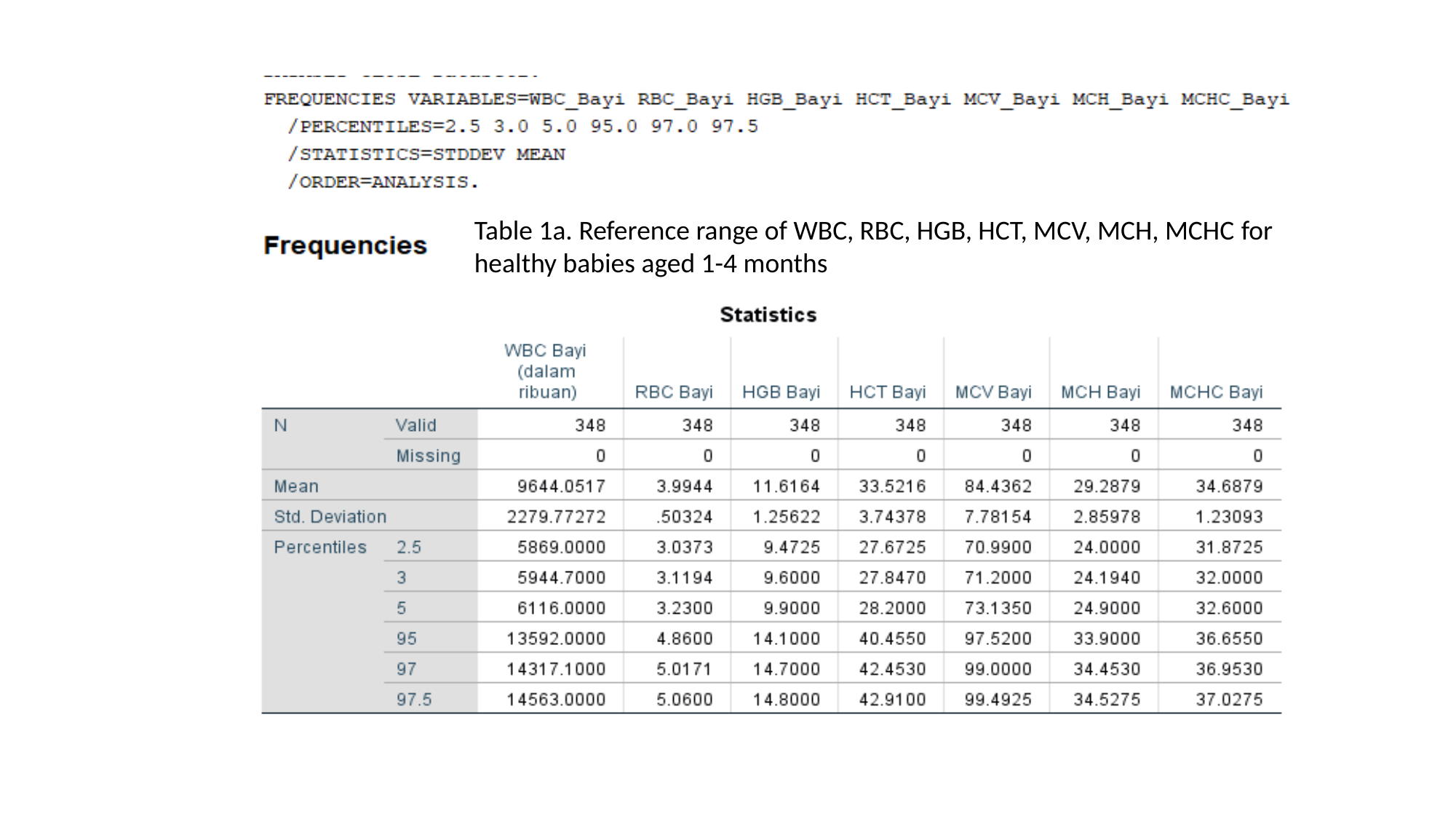

Table 1a. Reference range of WBC, RBC, HGB, HCT, MCV, MCH, MCHC for healthy babies aged 1-4 months

## Slide 2
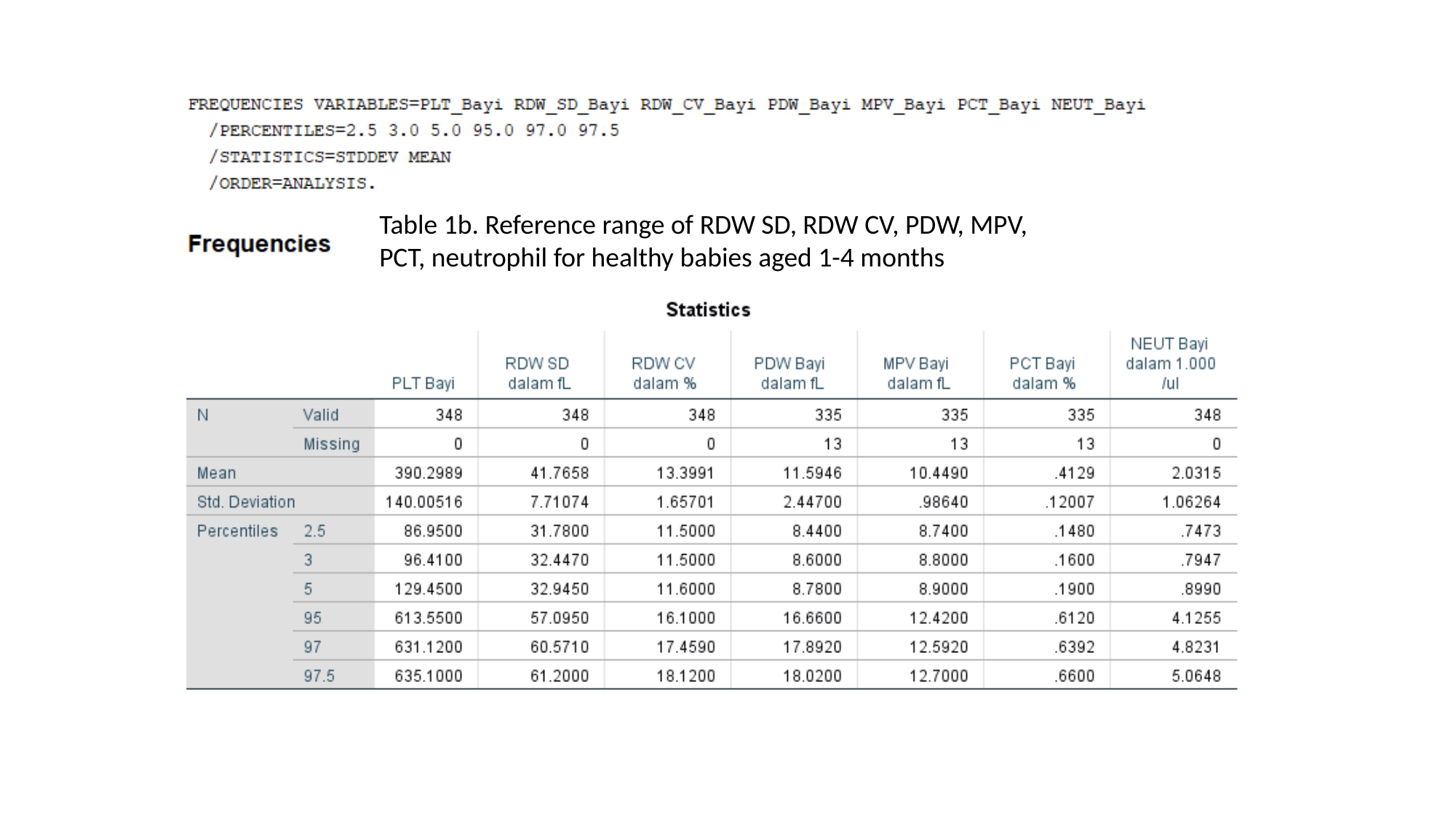

Table 1b. Reference range of RDW SD, RDW CV, PDW, MPV, PCT, neutrophil for healthy babies aged 1-4 months

## Slide 3
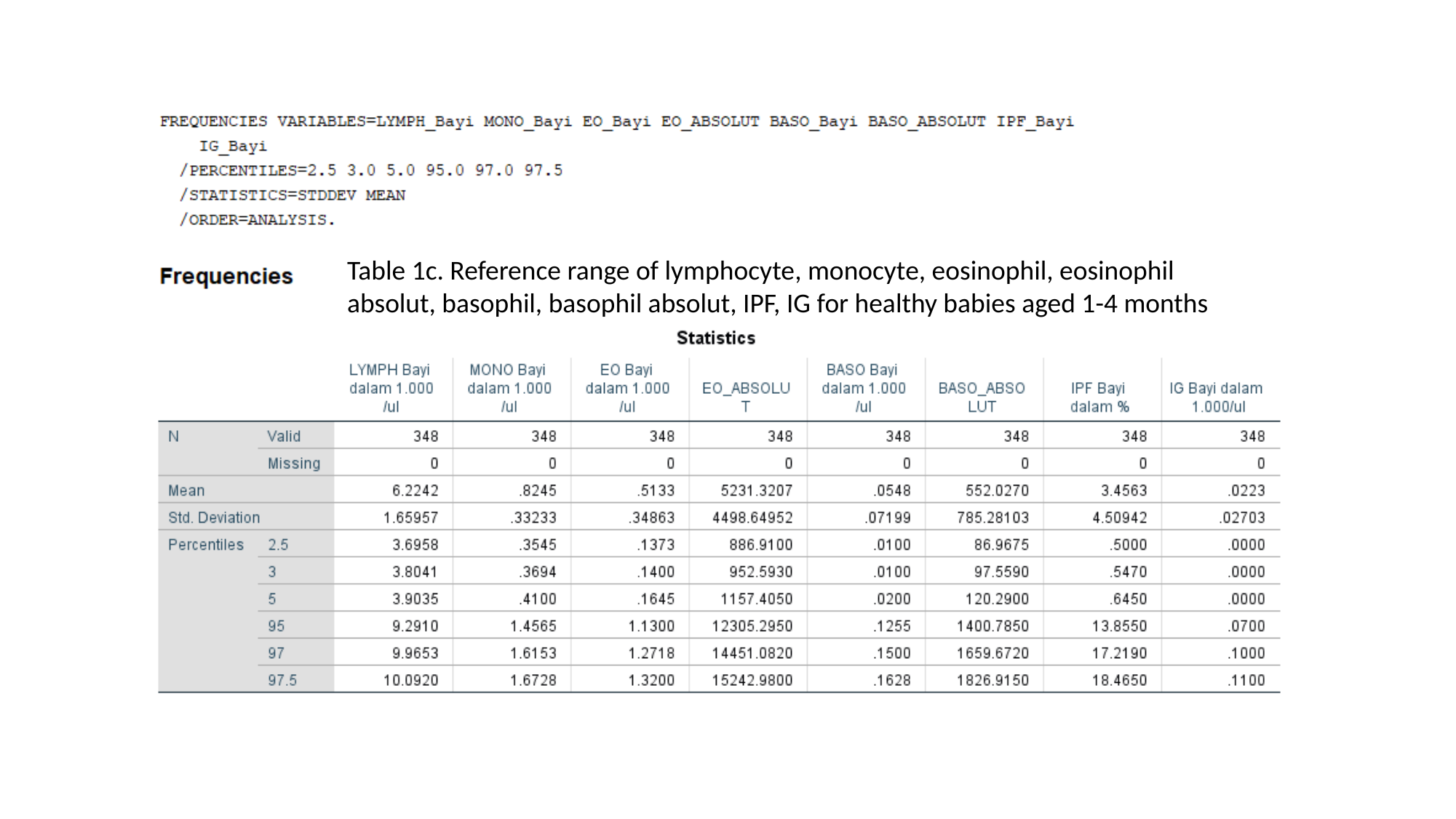

Table 1c. Reference range of lymphocyte, monocyte, eosinophil, eosinophil absolut, basophil, basophil absolut, IPF, IG for healthy babies aged 1-4 months

## Slide 4
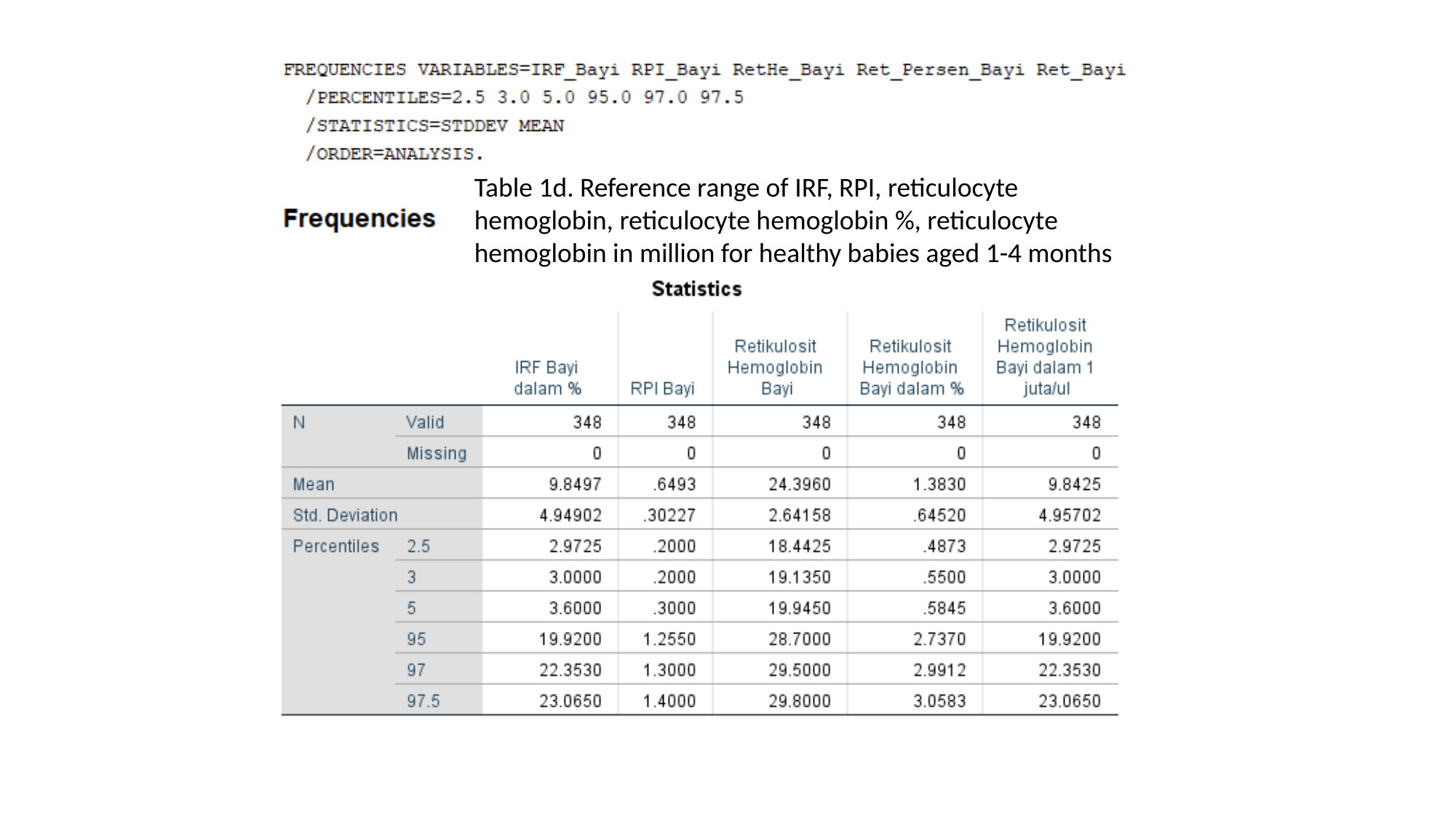

Table 1d. Reference range of IRF, RPI, reticulocyte hemoglobin, reticulocyte hemoglobin %, reticulocyte hemoglobin in million for healthy babies aged 1-4 months
