# Supplementary material for: Reference range of complete blood count, Ret-He, immature reticulocyte fraction, reticulocyte production index in healthy babies aged 1–4 months
Source: Sci Rep. 2023 Jan 9;13:423. doi: 10.1038/s41598-023-27579-3 (PMC9829736; doi:10.1038/s41598-023-27579-3)
Supplement: Supplementary file 4 — Supplementary Table 4. [file 41598_2023_27579_MOESM4_ESM.pptx]

## Slide 1
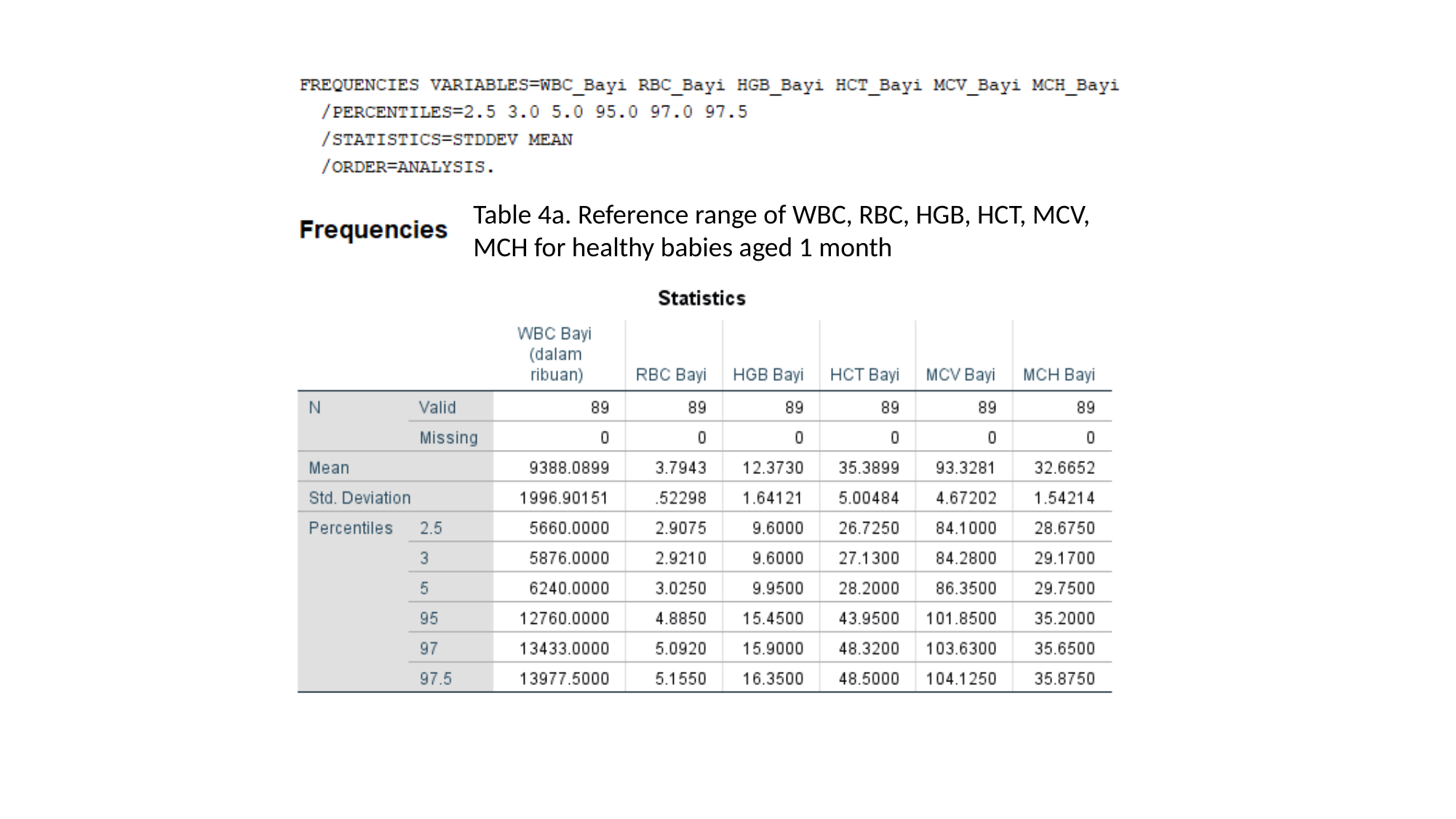

Table 4a. Reference range of WBC, RBC, HGB, HCT, MCV, MCH for healthy babies aged 1 month

## Slide 2
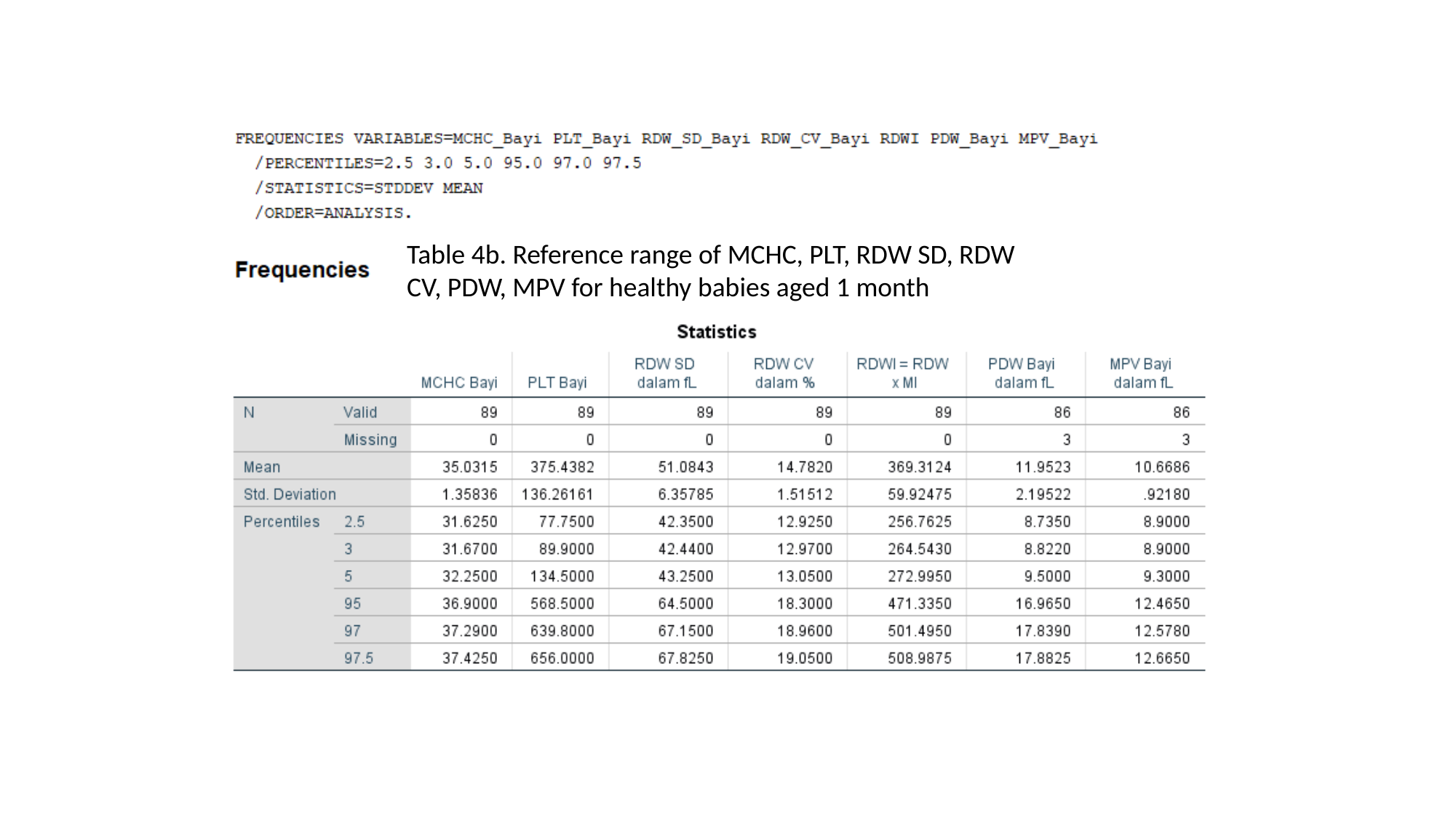

Table 4b. Reference range of MCHC, PLT, RDW SD, RDW CV, PDW, MPV for healthy babies aged 1 month

## Slide 3
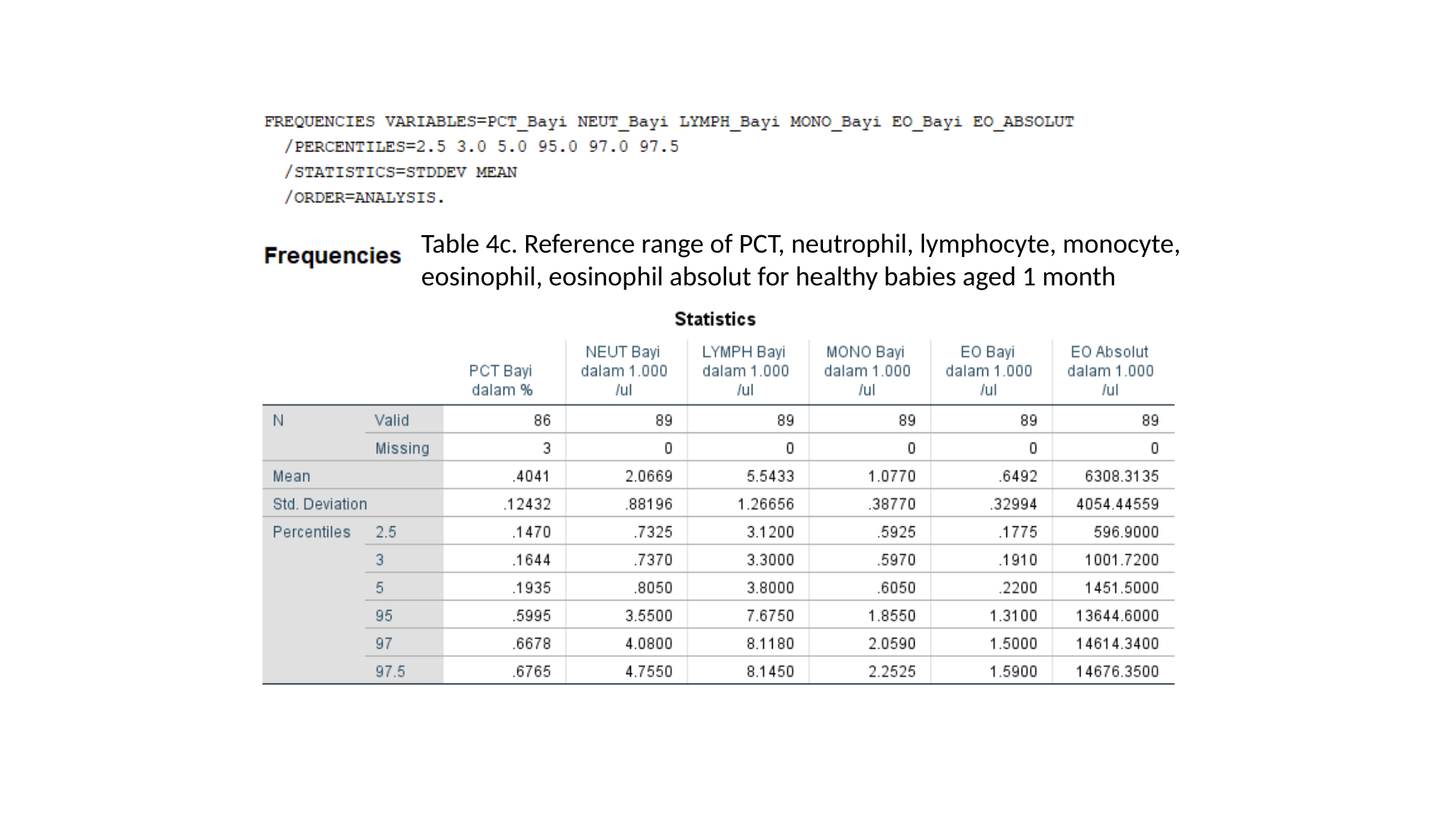

Table 4c. Reference range of PCT, neutrophil, lymphocyte, monocyte, eosinophil, eosinophil absolut for healthy babies aged 1 month

## Slide 4
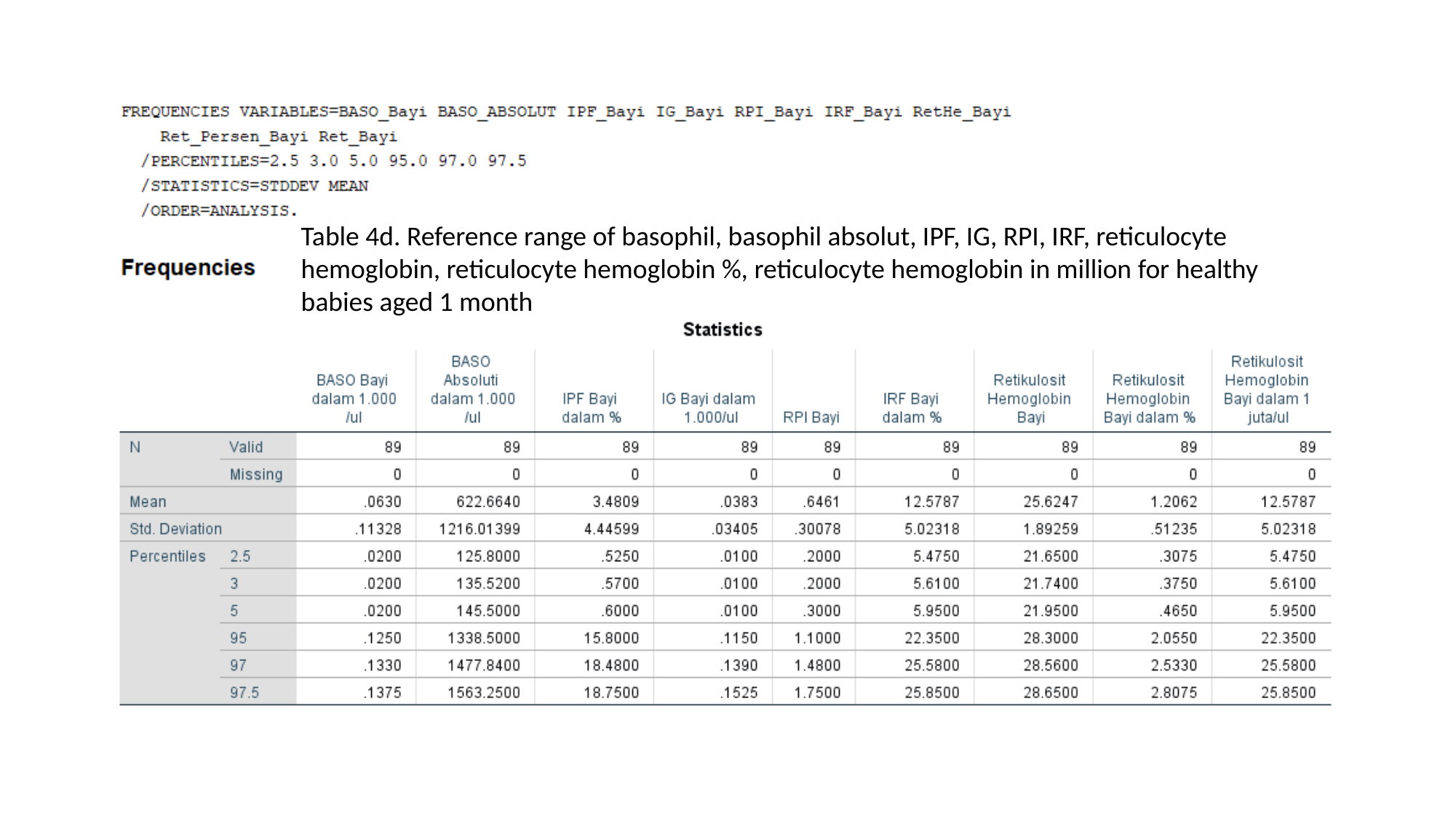

Table 4d. Reference range of basophil, basophil absolut, IPF, IG, RPI, IRF, reticulocyte hemoglobin, reticulocyte hemoglobin %, reticulocyte hemoglobin in million for healthy babies aged 1 month
