# Supplementary material for: Reference range of complete blood count, Ret-He, immature reticulocyte fraction, reticulocyte production index in healthy babies aged 1–4 months
Source: Sci Rep. 2023 Jan 9;13:423. doi: 10.1038/s41598-023-27579-3 (PMC9829736; doi:10.1038/s41598-023-27579-3)
Supplement: Supplementary file 5 — Supplementary Table 5. [file 41598_2023_27579_MOESM5_ESM.pptx]

## Slide 1
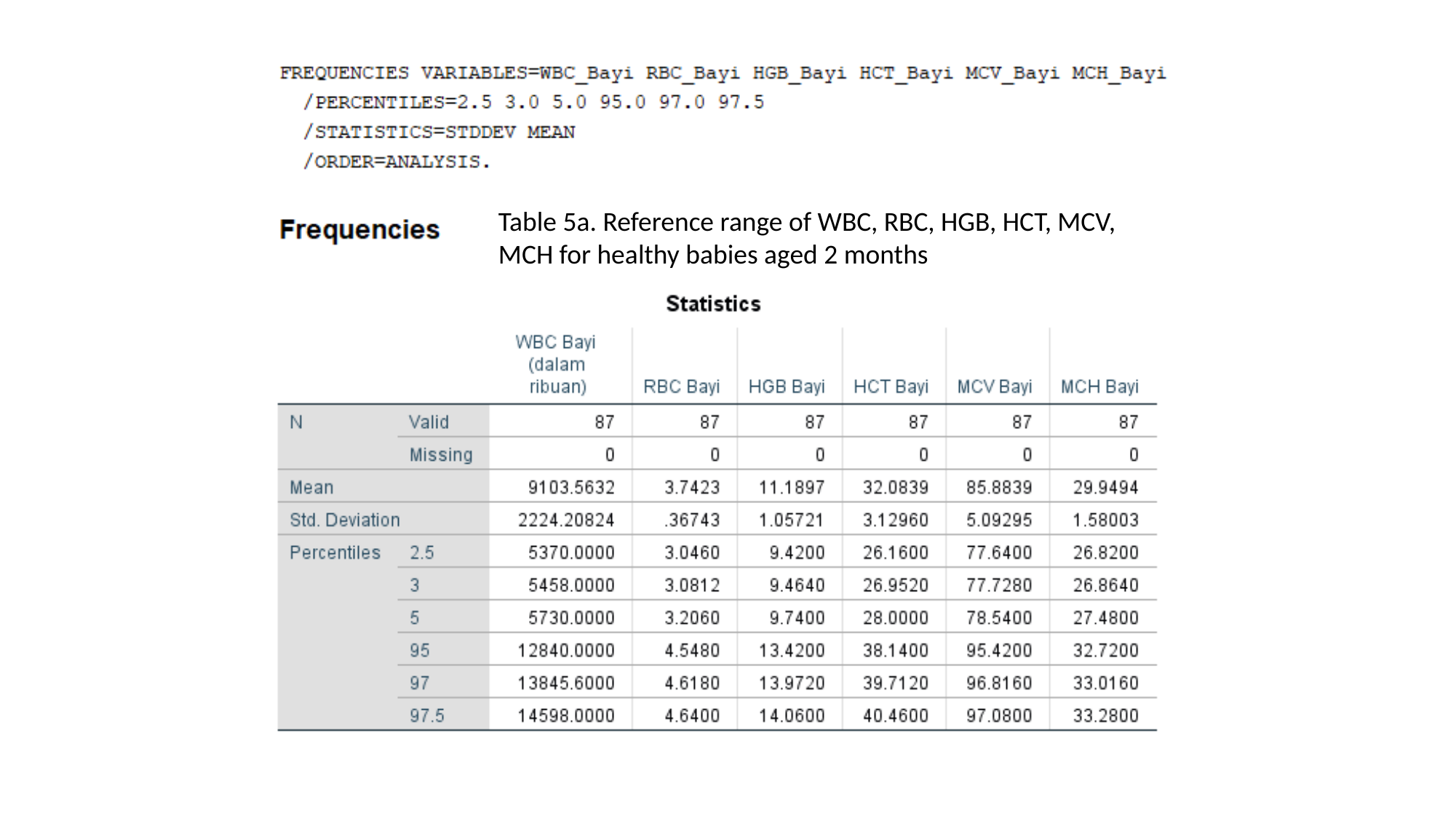

Table 5a. Reference range of WBC, RBC, HGB, HCT, MCV, MCH for healthy babies aged 2 months

## Slide 2
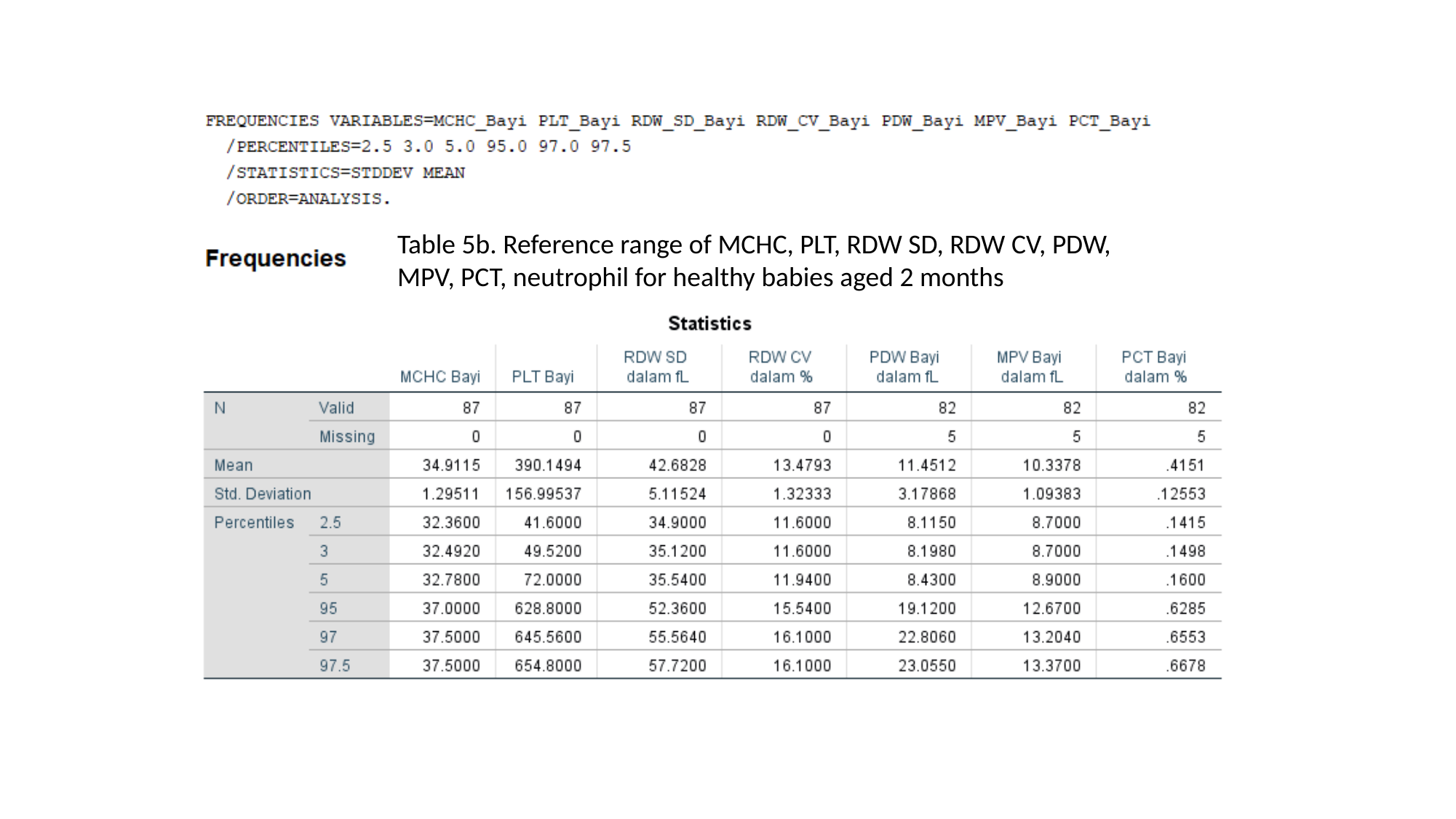

Table 5b. Reference range of MCHC, PLT, RDW SD, RDW CV, PDW, MPV, PCT, neutrophil for healthy babies aged 2 months

## Slide 3
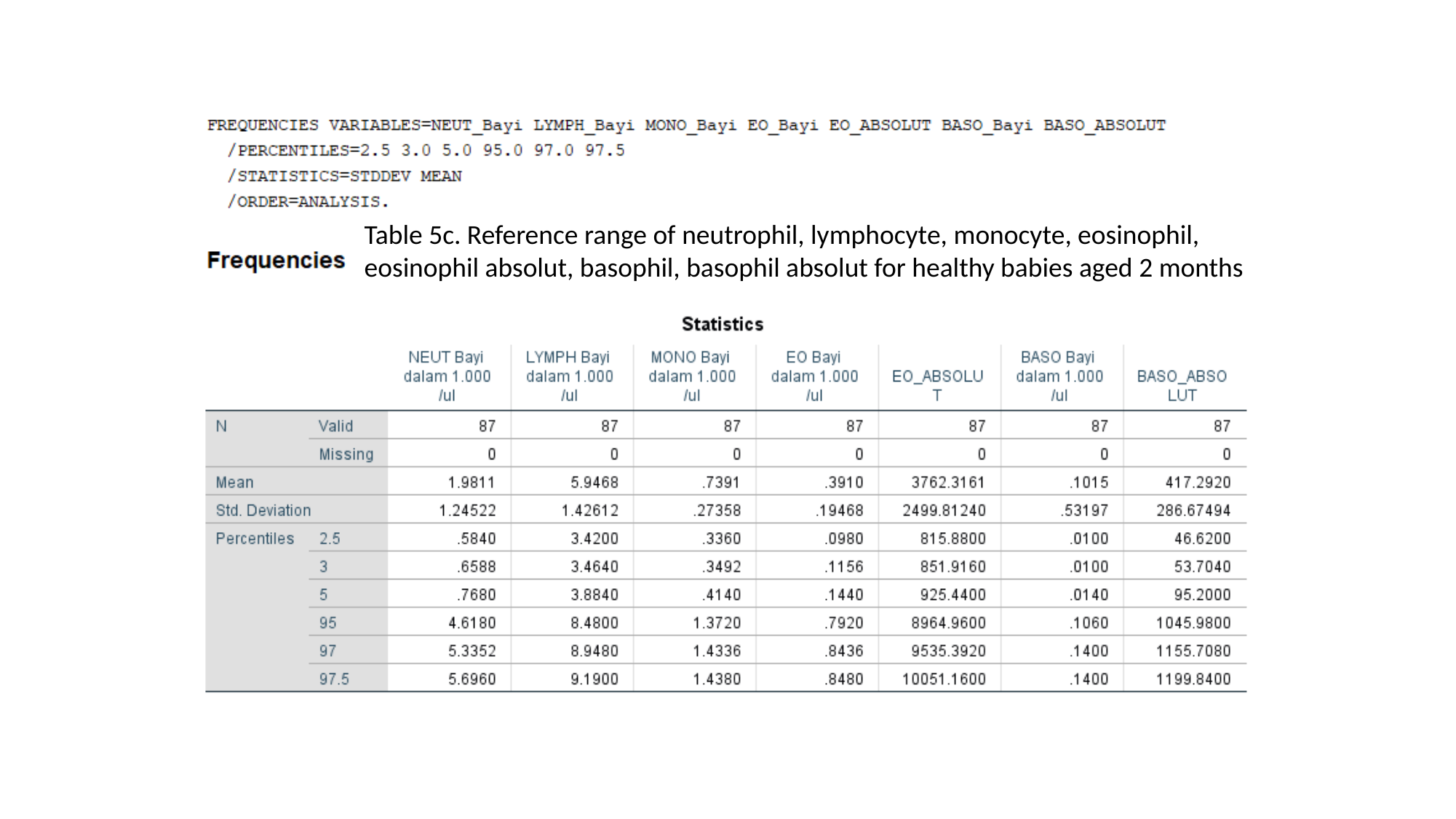

Table 5c. Reference range of neutrophil, lymphocyte, monocyte, eosinophil, eosinophil absolut, basophil, basophil absolut for healthy babies aged 2 months

## Slide 4
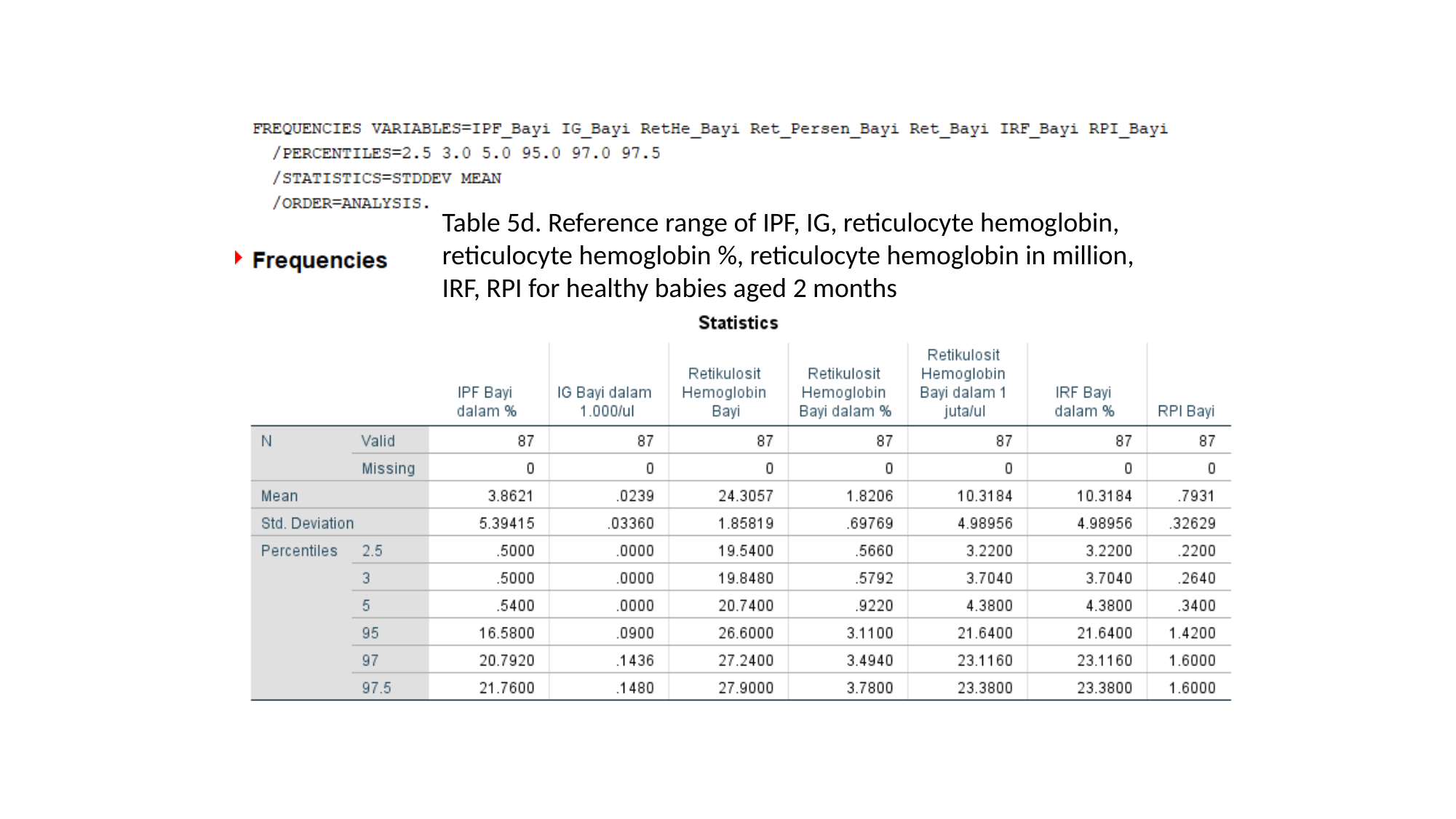

Table 5d. Reference range of IPF, IG, reticulocyte hemoglobin, reticulocyte hemoglobin %, reticulocyte hemoglobin in million, IRF, RPI for healthy babies aged 2 months
