# Supplementary material for: Reference range of complete blood count, Ret-He, immature reticulocyte fraction, reticulocyte production index in healthy babies aged 1–4 months
Source: Sci Rep. 2023 Jan 9;13:423. doi: 10.1038/s41598-023-27579-3 (PMC9829736; doi:10.1038/s41598-023-27579-3)
Supplement: Supplementary file 6 — Supplementary Table 6. [file 41598_2023_27579_MOESM6_ESM.pptx]

## Slide 1
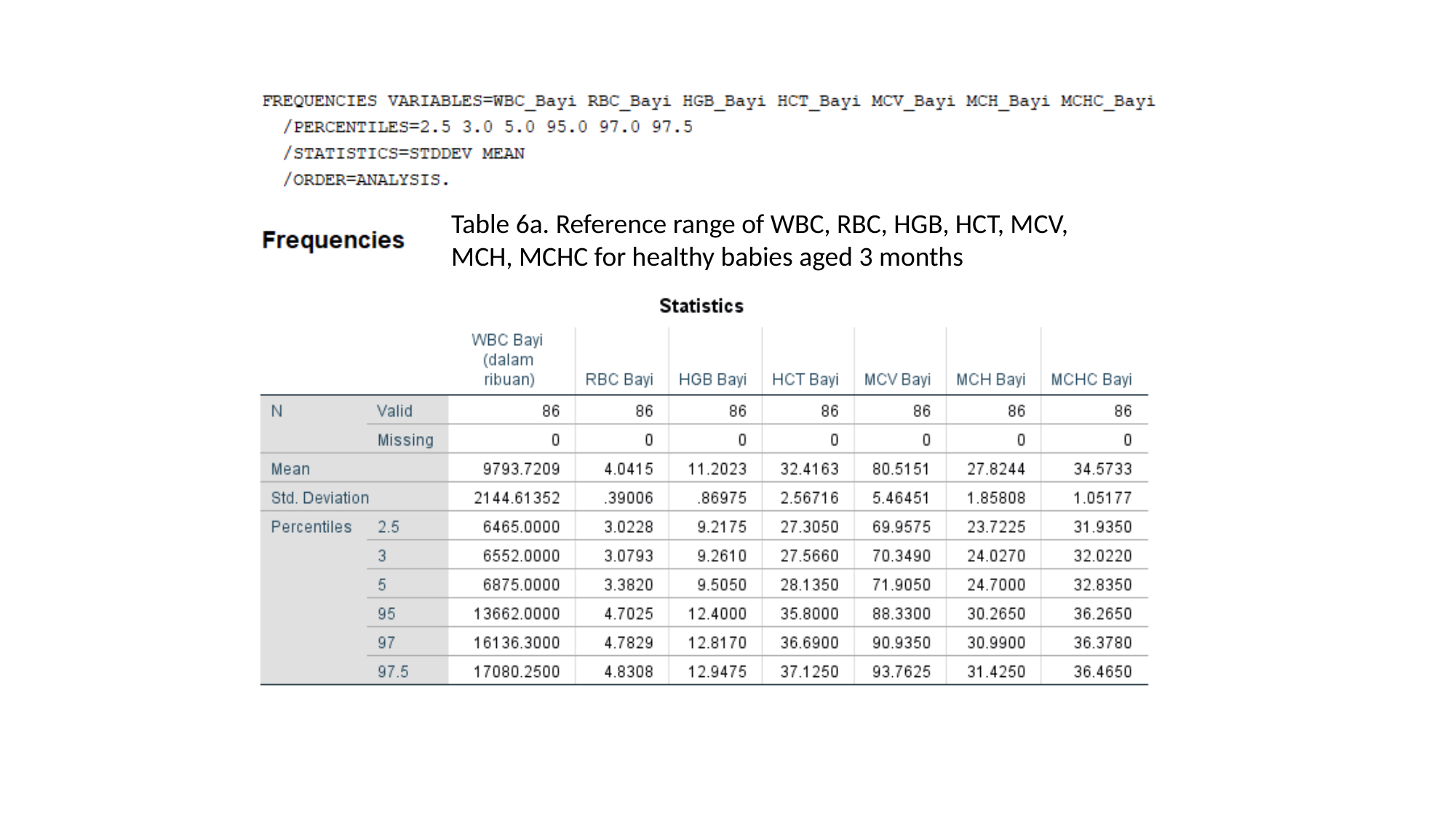

Table 6a. Reference range of WBC, RBC, HGB, HCT, MCV, MCH, MCHC for healthy babies aged 3 months

## Slide 2
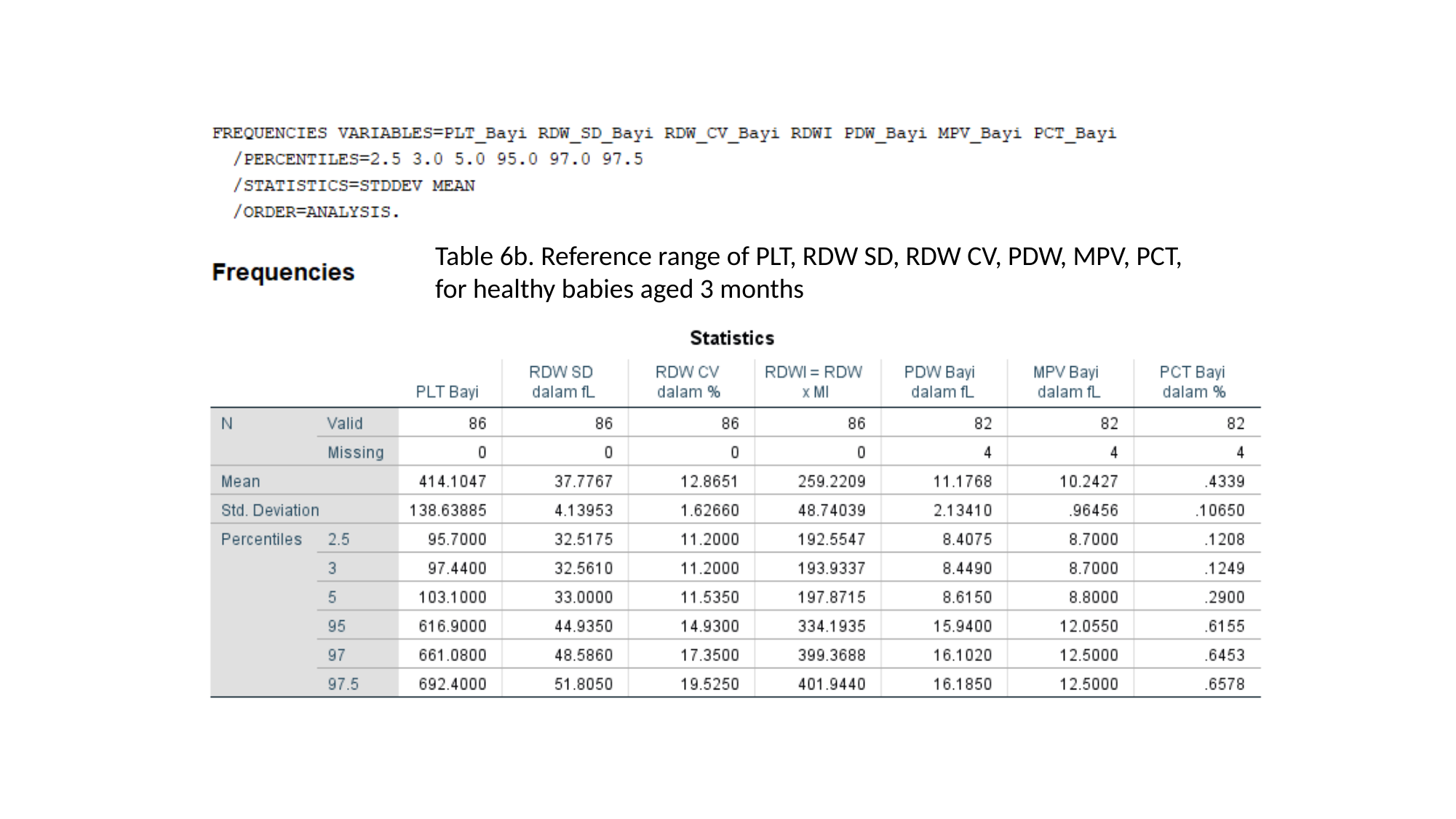

Table 6b. Reference range of PLT, RDW SD, RDW CV, PDW, MPV, PCT, for healthy babies aged 3 months

## Slide 3
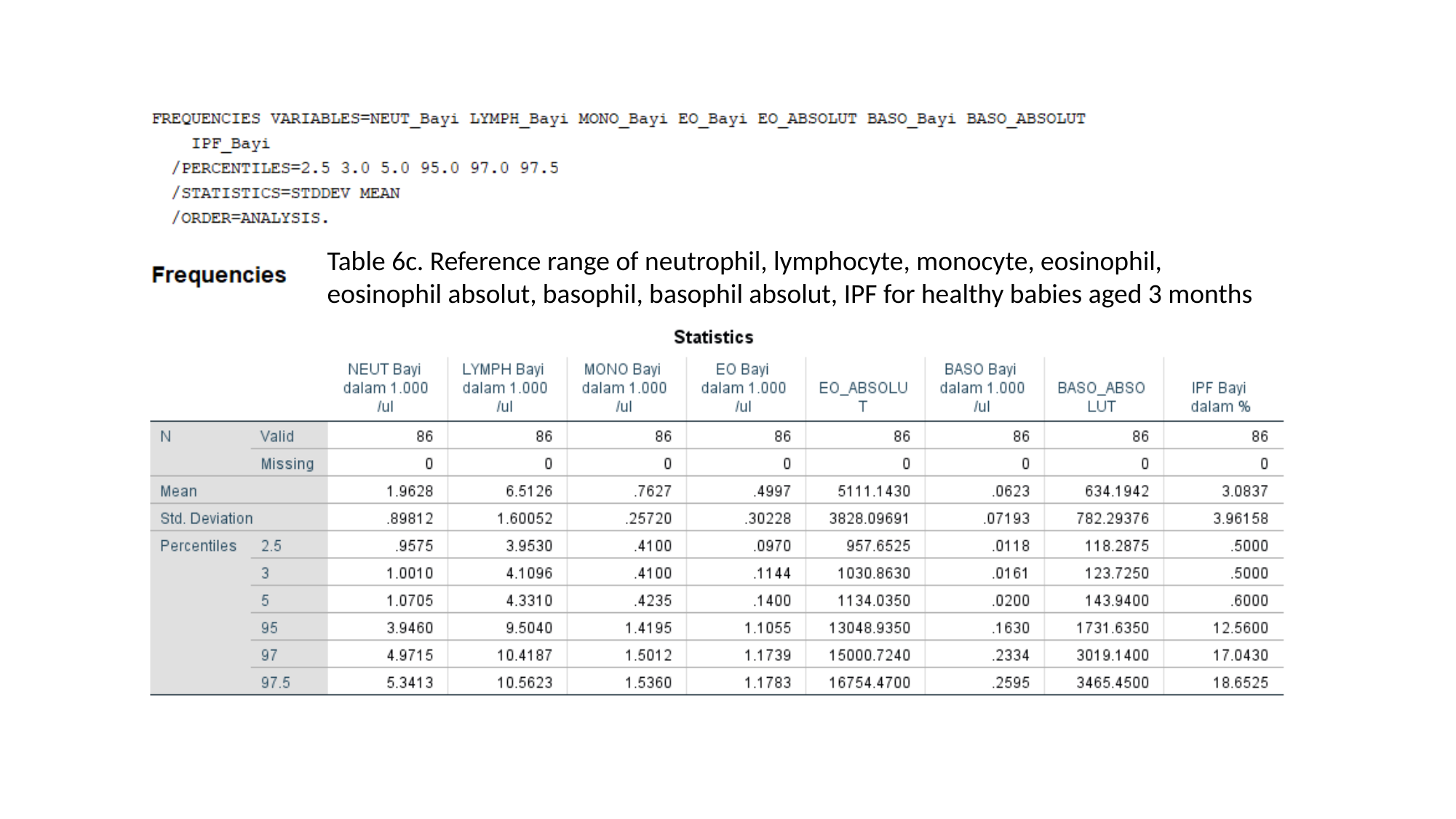

Table 6c. Reference range of neutrophil, lymphocyte, monocyte, eosinophil, eosinophil absolut, basophil, basophil absolut, IPF for healthy babies aged 3 months

## Slide 4
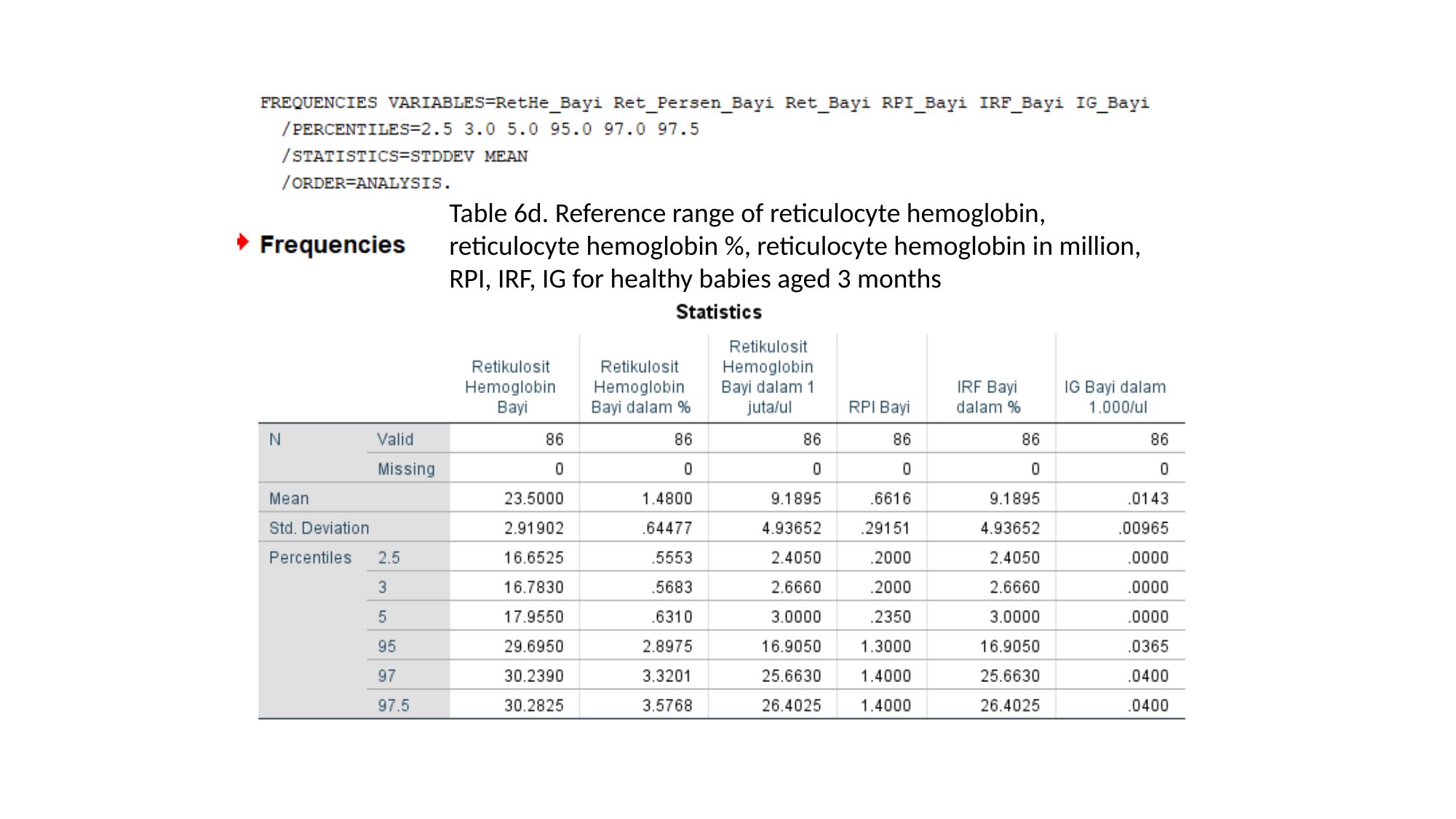

Table 6d. Reference range of reticulocyte hemoglobin, reticulocyte hemoglobin %, reticulocyte hemoglobin in million, RPI, IRF, IG for healthy babies aged 3 months
